# Cluster analysis of Canadian Armed Forces veterans living with chronic pain: Life After Service Studies 2016

**DOI:** 10.1080/24740527.2021.1898278

**Published:** 2021-04-21

**Authors:** Julian Reyes Velez, James M. Thompson, Jill Sweet, Jason W. Busse, Linda VanTil

**Affiliations:** a VAC Research Directorate, Charlottetown, Prince Edward Island, Canada; b Department of Public Health Sciences, Queens University, Kingston, Ontario, Canada; c Michael DeGroote Pain Centre, McMaster University, Hamilton, Ontario, Canada

**Keywords:** veterans, chronic pain, mental health, activity limitations, population study

## Abstract

**Objective**: This study explored the heterogeneity of Canadian Armed Forces veterans living with chronic pain to inform service needs planning and research using cluster analysis.

**Design**: We used a national cross-sectional Statistics Canada population survey.

**Participants**: Participants included 2754 Canadian Armed Forces (CAF) Regular Force veterans released from service between 1998 and 2015 and surveyed in 2016.

**Methods**: We used cluster analysis of veterans with chronic pain based on pain severity, mental health, and activity limitation characteristics. We compared clusters for sociodemographic, health, and service utilization characteristics.

**Results**: Of 2754 veterans, 1126 (41%) reported chronic pain. Veterans in cluster I (47%) rarely had severe pain (2%) or severe mental health problems (8%), and none had severe activity limitations. Veterans in cluster II (26%) more often than veterans in cluster I but less often than veterans in cluster III endorsed severe pain (27%) and severe mental health problems (22%) and were most likely to report severe activity limitation (91%). Veterans in cluster III (27%) were most likely to report severe pain (36%) and severe mental health problems (96%), and a majority reported severe activity limitations (72%). There was evidence of considerable heterogeneity among individuals in terms of socioeconomic characteristics, pain characteristics, mental and physical health status, activity limitations, social integration, and service utilization indicators.

**Conclusions**: About half of Canadian veterans living with chronic pain infrequently endorse severe pain or serious mental health issues without severe activity limitations. The other half had more complex characteristics. The heterogeneity of CAF veterans with chronic pain emphasizes the need for support systems that can address variability of needs.

## RÉSUMÉ

**Objectif**: Cette étude portait sur l’hétérogénéité des anciens combattants des Forces armées canadiennes vivant avec la douleur chronique pour éclairer la planification et la recherche en matière de besoins de services à l’aide de l’analyse par groupes.

**Devis**: Nous avons utilisé une enquête nationale transversale sur la population de Statistique Canada.

**Participants**: Les participants comprenaient 2 754 anciens combattants de la Force régulière des Forces armées canadiennes (FAC) libéré du service entre 1998 et 2015 et enquêtés en 2016.

**Méthodes**: Nous avons utilisé une analyse par groupes d’anciens combattants souffrant de douleur chronique fondée sur l’intensité de la douleur, la santé mentale et les caractéristiques en matière de limitation d’activité. Nous avons comparé les caractéristiques sociodémographiques, de santé et d’utilisation des services des groupes.

**Résultats**: Sur 2 754 anciens combattants, 1 126 (41 %) ont fait état d’une douleur chronique. Les anciens combattants du groupe I (47 %) avaient rarement une douleur intense (2 %) ou de graves problèmes de santé mentale (8 %), et aucun d’entre eux n’avait de limitation d’activité sévère. Les anciens combattants du groupe II (26%) souffraient de douleur intense (27 %) et de problèmes de santé mentale graves (22 %) plus souvent que les anciens combattants du groupe I mais moins souvent que les anciens combattants du groupe III et étaient plus susceptibles de déclarer une limitation d’activité sévère (91 %). Les anciens combattants du groupe III (27 %) étaient les plus susceptibles de déclarer une douleur intense (36 %) et des problèmes de santé mentale graves (96 %), et la majorité d’entre eux a signalé une limitation d’activité grave (72%). Les données probantes ont révélé une hétérogénéité considérable parmi les individus en ce qui concerne les indicateurs relatifs aux caractéristiques socioéconomiques, aux caractéristiques de la douleur, à l’état de santé mentale et physique, à la limitation d’activité, à l’intégration sociale et à l’utilisation des services.

**Conclusions**: Environ la moitié des anciens combattants canadiens vivant avec une douleur chronique souffrent rarement de douleur intense ou de problèmes de santé mentale graves sans avoir de limitations d’activité graves. L’autre moitié avait des caractéristiques plus complexes. L’hétérogénéité des vétérans des FAC souffrant de douleur chronique souligne l’importance que des systèmes de soutien capables de répondre à la diversité des besoins soient disponibles.

## Introduction

Chronic pain is an important public health issue, affecting approximately one in five people in the general population in Canada and worldwide.^1,2^ In the Life After Service Studies (LASS) surveys, the prevalence of chronic pain among Canadian Armed Forces (CAF) Regular Force veterans (former members) released since 1998 was estimated at 41% to 64% depending on the self-report question used, which is at least double the prevalence in the comparable Canadian general population (21% in 2016).^3,4^ Chronic pain is more prevalent in military veterans than in general populations for reasons that remain unclear, in part because military personnel are exposed to rigorous training and hazardous activities where injuries are more likely to occur.^4–10^ Chronic pain is common in serving CAF personnel and is a common reason for early release from service, as in the United States.^3,5,6,9–11^

The transition from military service to post-service life can be difficult to navigate, which can lead to adverse well-being outcomes.^12,13^ This is particularly true for veterans living with chronic health conditions, including chronic pain.^14,15^ In the LASS surveys, CAF veterans released since 1998 were found to have a higher prevalence of both chronic physical and mental health conditions than the comparable Canadian general population, including painful conditions such as arthritis, back pain, bowel disorders, and migraine.^3,16,17^ Regular Force and some Reserve Force CAF members obtain health care through CAF Health Services while serving, but like many serving Reserve Force members and after release, they must find health care providers in civilian health care systems where their military experience might not be fully understood. Finding solutions to providing veteran-centric care is therefore a priority.

Challenges in meeting the needs of people with chronic pain are universal: European studies have shown that though around 20% of the adult population experiences chronic pain, less than 2% are managed with specialized care.^7^ In Canada, as elsewhere, lack of clarity in best practice care pathways tailored to the needs of people with chronic pain hampers support planning, education, health care efficiencies, and outcomes.^2^ Planning services to assist veterans living with chronic pain requires an understanding of the extent and nature of heterogeneity in terms of physical and mental health, pain severity, activity limitations, comorbidity, and well-being support needs at different stages of life.^1,11,18,19^ Particularly for those also affected by psychiatric disorders, chronic pain is an important driver of health care utilization in primary and other health care services.^6,11,18,19^ In the LASS surveys, veterans with diagnosed mental health conditions very frequently had co-occurring chronic physical health conditions often characterized by pain.^17^ Veterans with co-occurring physical and mental health conditions were synergistically more likely to have activity limitations or need for assistance with daily living than those with none or one or the other.^20^

Heterogeneity challenges planning for support services for veterans living with chronic pain, often requiring coordinated access to a several health care providers and other support services.^2,18,21^ Integrated, person-centered approaches that can match the type and intensity of supports to individual needs and goals are thought to be optimal.^2,22^ In 1998, the U.S. Veterans Health Administration adopted a veteran-centered, interdisciplinary, multimodal national pain management strategy.^23^ A variety of options for organizing care have been proposed, including stepped, stratified, and matched care models.^2,22^ Although there is emerging evidence for various approaches,^2,22,24^ no consensus has been achieved in Canada, each has disadvantages, and there are considerable systemic barriers to organizing chronic pain care.^2,18,22,25^ Though there is some basic epidemiologic information about chronic pain prevalence and correlates in CAF serving members and veterans,^5,8,26,27^ there is no assessment of heterogeneity in this population to support policy development, programming, research, and service model development.

In 2019, Veterans Affairs Canada (VAC) announced significant funding for a Center of Excellence for Chronic Pain for Canadian Veterans.^28^ The Center’s areas of focus include evidence-based care guidelines and research. To support the Center’s work and planning by agencies providing care to veterans, our objective was to explore the extent and nature of heterogeneity among CAF veterans living with chronic pain who were released since 1998 using cluster and descriptive analysis of cross-sectional LASS data.

## Methods

### Study Design and Population

The LASS 2016 survey was a cross-sectional computer-assisted telephone interview survey of the well-being of CAF Regular Force veterans released between 1998 and 2015 and surveyed in February to March 2016.^4^

The survey had a response rate of 73%, and 91% of respondents agreed to allow Statistics Canada to share data with VAC for research analyses. The sample size of 2754 represented a weighted population of 56,420 veterans who, at the time of the survey, had not reenrolled in the CAF and were not living in institutions, the territories (Northwest Territories, Nunavut, or Yukon), or outside of Canada. Veterans released at entry ranks of second lieutenant, acting sub-lieutenant, and private recruit were excluded to focus on those who had completed initial training. The survey was stratified by rank at release and included questions from national Canadian population health studies covering multiple domains of well-being.^4^ Veteran status and military characteristics were obtained from the Department of National Defense human resource database, and other indicators were self-reported in the survey.

### Ethics and Data Privacy

Data collection was funded by VAC and conducted by Statistics Canada, using computer-assisted telephone interviews. The survey data are available for approved projects through the Research Data Center Network (crdcn.org). This article uses anonymous data from the 91% of respondents who agreed to share with VAC. Data access procedures for the survey were reviewed and approved by the relevant policy committees at Statistics Canada that fulfill the functions of a research ethics board.

### Chronic Pain, Pain Intensity, and Number of Activities Prevented by Pain

Veterans living with chronic pain were identified using the Health Utilities Index Mark 3 (HUI3) chronic pain module question: “Are you usually free of pain or discomfort?”^4^ Those answering “no” were included in the analysis of chronic pain clusters. Pain intensity was assessed with “How would you describe the usual intensity of your pain or discomfort?” (response options: mild, moderate, or severe). Number of activities prevented by pain or discomfort was assessed with “How many activities does your pain or discomfort prevent?” (response options: none, a few, some, or most). The utility of the module was assessed for the general Canadian population and has been used previously in general population surveys.^29^

### Socioeconomic and Military Characteristics

Socioeconomic characteristics (age, sex, education attained, marital status, household income, satisfaction with finances, and main activity) were obtained with self-reported questions used in the Canadian Community Health Surveys (CCHS). Military characteristics were obtained by data linkage to respondents’ information.

### Physical and Mental Health

Questions about chronic diagnosed physical and mental health conditions were taken from the CCHS.^4^ Following the preamble, “We are interested in conditions diagnosed by a health professional and are expected to last or have already lasted 6 months or more,” respondents were asked about several sentinel chronic conditions. Self-reported hearing problems were assessed with the HUI3 hearing module.^30^ The physical health conditions were combined by organ system for this analysis. The specific mental health condition questions posed to respondents were (1) “Do you have a mood disorder such as depression, mania, dysthymia, or bipolar disorder?”; (2) “Do you have an anxiety disorder such as a phobia, obsessive–compulsive disorder, or a panic disorder?”; and (3) “Do you have posttraumatic stress disorder (PTSD)?”

Past-month psychological distress symptoms potentially attributable to depression and anxiety were measured with the Kessler Psychological Distress Scale (K-10). The total score for the K-10 ranges from 0 to 40, with higher scores indicating greater psychological distress. Cutoffs were adopted from Canadian and Australian studies based on studies in general and military populations: 0–9 for no/little distress, 10–19 for mild/moderate distress, and 20–40 for severe stress.^17,31,32^

PTSD symptoms were measured with the *Diagnostic and Statistical Manual of Mental Disorders*, 4th edition version of the Primary Care Posttraumatic Stress Disorder (PC-PTSD) screener to assess for possible PTSD in three categories: none; one or two of four criteria; or three or four of four criteria. Validation in U.S. Veterans Health Administration (VHA) patients found that the optimally efficient cutoff for detecting possible PTSD was three criteria.^33,34^ Because subthreshold PTSD can be associated with significant impairment, we also reported the proportion of respondents who endorsed one to two PC-PTSD criteria.^32^

The composite measure of mental health problems developed for analysis of LASS survey data combined the categories of K-10 and PC-PTSD symptom measures and absence/presence of self-reported diagnosed chronic mental health conditions (mood disorders, anxiety disorders, or PTSD) yielding three levels of severity: no/little, moderate, and severe.^32^ Those with low K-10 scores, no PC-PTSD criteria, and no diagnosed mental health condition were categorized as no or little mental health problems. Those with a high K-10 score or presence of three or four PC-PTSD criteria were categorized as severe, and the rest were mild–moderate, whether they had a diagnosed chronic condition or not. The measure correlated in expected ways with well-being characteristics and service use in LASS surveys.^32^

## Health-related Quality of Life

Health-related quality of life was measured using the 12-item Short Form Health Survey (SF-12). Physical and Mental Component Summary (PCS, MCS) scores were computed using QualityMetric software.^52^ The software computes summary scores for individuals based on normative data for the 1998 U.S. noninstitutionalized general population. Canadian population norms are only slightly higher.^3^ PCS and MCS scores are transformed and standardized to a mean of 50 and a standard deviation of 10, with scores above and below 50 indicating better or poorer than average health-related quality of life (HRQoL), respectively. Lower SF-12 scores indicate lower HRQoL in a nonlinear manner: 98% of the reference population has better HRQoL than those with scores of 30 or less, and 84% have better HRQoL than those with scores of 40 or less.

### Life Stress and Suicidal Ideation

Life stress was assessed with the question “Thinking about the amount of stress in your life, would you say that most days are … not at all, not very, a bit, quite a bit, extremely (stressful)?” Past-year suicidal ideation was assessed with “Have you ever seriously considered committing suicide or taking your own life?” followed by “Has this happened in the past 12 months”?

### Activity Limitations

A three-category indicator of activity limitations, a proxy for role participation disability, was derived in prior LASS analyses using two questions from the CCHS: (1) whether a long-term physical or mental condition or health problem reduced the amount or kind of activity at home, school, work, or other, sometimes or often (health-related activity limitations), and (2) need for assistance with at least one basic or instrumental activity of daily living (ADL need).^20^

### Perceived Ease of Adjustment to Civilian Life

Perceived ease of adjustment to civilian life was assessed with the survey question “In general, how has the adjustment to civilian life been since you were released from the Canadian Armed Forces?” The five options were combined into three categories: (1) very/moderately easy; (2) neither easy nor difficult; or (3) very/moderately difficult. This indicator was adapted from a U.S. Air Force study and was found in prior LASS analyses to be correlated with well-being indicators.^35^

### Social Support and Service Utilization

Perceived social support was measured using the Social Provisions Scale, with scores ranging from 10 to 40. The cutoff for low social support was <30 based on prior LASS analysis.^26^ The survey asked about whether the respondent had seen or talked to a health professional about emotional or mental health, whether they had been a patient overnight in hospital, and number of family doctor visits. VAC clients were those respondents in receipt of benefits at the time of the survey, based on data linkage.

### Well-being

In this article, *well-being* refers to the conceptual framework used at VAC in which well-being is viewed as a superordinate concept described subjectively and objectively across seven subordinate domains: health, employment or other meaningful activity, finances, life skills/preparedness, social integration, housing/physical environment, and cultural/social environment. In this framework, well-being fluctuates across the life course in response to interconnected determinants from all domains.^26,36,37^

### Analysis

We used descriptive statistics to calculate chronic pain prevalence and frequencies of characteristics of veterans with and without chronic pain, using weighted estimations. Pearson’s chi-square was used to test the relationship between pain and the independent variables of each domain. Two-sided tests of equality for column means were assessed for continuous variables in SPSS v25 for Windows (IBM Corp., Armonk, NY).^53^ Bootstrap weights provided by Statistics Canada were applied to convert unweighted frequencies to represent the Canadian veteran population and adjust for nonresponse bias and the survey sampling design.^4^ We calculated the associated 95% confidence intervals (CIs) using Taylor linearization with Stata v13.1 (StataCorp, College Station, TX).^54^

**Figure 1.**
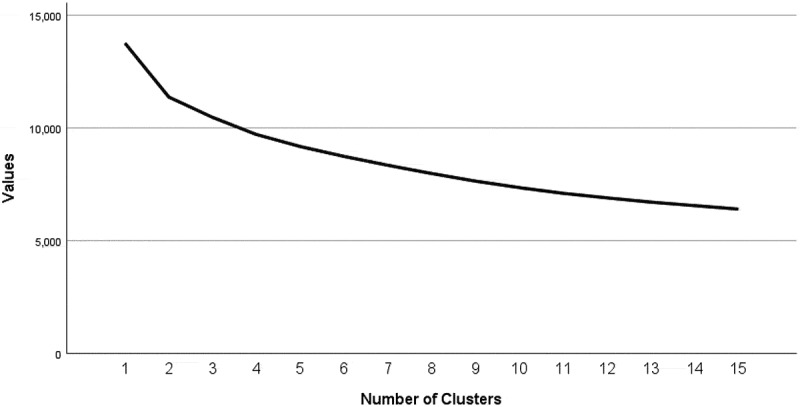
Autoclustering AIC values by number of clusters

We conducted two-step cluster analysis for veterans living with chronic pain to explore subgroups. We chose five classification variables based on similar studies in chronic pain populations and assessment of prior literature about factors that can distinguish case complexity: (1) pain intensity, (2) number of activities prevented by pain, (3) K-10 psychological distress, (4) PC-PTSD symptoms, and (5) health-related activity limitations.^1,11,18,19,38–44^ We used the two-step cluster analysis subroutines in SPSS v25 for Windows.^55^ The software used an agglomerative hierarchical method accommodating both continuous and categorical attributes. Initially, the automated clustering produced a two-cluster solution that we did not find informative. We used Akaike’s information criterion (AIC) graphical assessment to look for the most appropriate number of clusters, together with the average silhouette coefficient measuring the separation of the clusters for the selected classification variables (Figure 1). The AIC graphical assessment showed other cluster solutions with lower AIC values; however, this posed a challenge for interpretability with no gain in the separation of the characteristics. The three-cluster solution had an optimum of both low AIC values and good interpretability with a moderate ratio of cluster sizes (1.8). The relative contribution of each classification variable to the cluster analysis was determined by a measure of predictor importance assessed by the SPSS two-step cluster module.

Finally, we examined the socioeconomic, military, health status, and service utilization characteristics of veterans in each cluster. Weights that were developed for the entire sample were not appropriate for use with the subpopulation, so we used sample data in the cluster analysis and subsequent descriptive analysis of the clusters. Statistical testing for differences between the clusters was assessed using pairwise tests of the equality of the clusters (columns) with Bonferroni correction in SPSS v25 for Windows.^56^ The test compares pairs within a category of a variable, taking into consideration the distribution of frequencies within each of the two columns of categories within each variable.

## Results

There were 1261 veterans who reported living with chronic pain, for a weighted prevalence of 40.7% (95% CI, 38.3–43.2).

### Comparison of Veterans with and without Chronic Pain

Table 1 compares veterans with and without chronic pain. Veterans with or without chronic pain had a mean age in the late 40s. Those with chronic pain were slightly older than those without pain: mean 49.7 years (95% CI, 48.9–50.4 years) versus 46.9 years (95% CI, 46.1–47.6) and more likely to be female. Veterans with chronic pain reported lower household incomes and were more likely to be dissatisfied with their finances than veterans without chronic pain. Moreover, veterans with chronic pain were less likely to be working and were more likely to be on disability than those without pain. Senior noncommissioned member rank and longer years of service were associated with chronic pain. Those with pain more often had physical and mental health problems, extreme life stress, suicidal ideation, and difficult adjustment to civilian life. Nearly three-quarters of those living with chronic pain were VAC clients as opposed to only a quarter of those without pain.

**Table 1.** Characteristics of veterans with and without chronic pain (weighted data)

		With pain		No pain		Total	
Characteristic	Category	Wt%	95% CI	Wt%	95% CI	Wt%	95% CI
Age***	<30	3.5	(2.4, 5.0)	10.5	(8.6, 12.8)	7.7	(6.4, 9.1)
	30–39	14.8	(11.9, 18.4)	21.6	(18.5, 25.0)	18.8	(16.6, 21.2)
	40–49	22.5	(19.5, 25.8)	20.1	(17.5, 23.1)	21.1	(19.1, 23.3)
	50–59	44	(40.4, 47.7)	31.3	(28.4, 34.3)	36.5	(34.2, 38.8)
	60+	15.2	(13.2, 17.4)	16.5	(14.7, 18.5)	16	(14.7, 17.4)
Sex**	Male	85.3	(82.3, 87.8)	89.6	(87.4, 91.5)	87.8	(86.1, 89.4)
	Female	14.7	(12.2, 17.7)	10.4	(8.5, 12.6)	12.2	(10.6, 13.9)
Education**	High school or less	47.7	(43.9, 51.4)	44.5	(41.2, 47.9)	45.8	(43.3, 48.3)
	Postsecondary < bachelor	39.9	(36.3, 43.7)	34.9	(31.7, 38.3)	36.9	(34.5, 39.4)
	Postsecondary ≥ bachelor	12.4	(10.6, 14.5)	20.6	(18.5, 22.9)	17.3	(15.9, 18.7)
Marital status	Married/common law	78.1	(74.7, 81.1)	76	(73.0, 78.8)	76.8	(74.6, 78.9)
	Separated/widowed/divorced	10.5	(8.5, 12.7)	10.5	(8.6, 12.8)	10.5	(9.1, 12.1)
	Single/never married	11.5	(9.1, 14.5)	13.5	(11.3, 16.0)	12.7	(11.0, 14.6)
Household income***	$0 to <$50,000	16.1	(13.3, 19.3)	11.1	(9.1, 13.5)	13.2	(11.5, 15.0)
	$50,000 to <$100,000	40.5	(36.8, 44.3)	36.3	(33.0, 39.8)	38	(35.5, 40.6)
	$100,000 to <$150,000	26.7	(23.4, 30.3)	23.6	(20.9, 26.6)	24.9	(22.7, 27.2)
	$150,000+	16.7	(13.9, 19.9)	28.9	(25.9, 32.2)	23.9	(21.8, 26.2)
Satisfaction with finances***	Very satisfied/satisfied	63	(59.3, 66.4)	72.7	(69.4, 75.7)	68.7	(66.3, 71.0)
	Neither	13.7	(11.5, 16.1)	13.7	(11.5, 16.3)	13.7	(12.1, 15.5)
	Dissatisfied/very dissatisfied	23.4	(20.3, 26.8)	13.6	(11.3, 16.4)	17.6	(15.7, 19.7)
Main activity***	Working or reserves	53.6	(50.0, 57.3)	75.3	(72.6, 77.8)	66.4	(64.2, 68.6)
	Retired, not looking for work	18.4	(16.0, 21.1)	14.9	(13.1, 16.9)	16.3	(14.9, 17.9)
	School or training	5.7	(4.2, 7.7)	3.7	(2.7, 5.2)	4.6	(3.6, 5.7)
	Looking for work	2.8	(2.0, 3.8)	1.7	(1.2, 2.6)	2.2	(1.7, 2.8)
	Caregiving family member	F	F	F	F	F	F
	Disabled or on disability	17	(14.4, 19.9)	2.2	(1.5, 3.3)	8.2	(7.0, 9.6)
Rank***	Officer	13.5	(12.0, 15.1)	20	(18.6, 21.6)	17.4	(16.6, 18.1)
	Senior NCM	36.2	(33.7, 38.9)	26.6	(24.6, 28.6)	30.5	(29.5, 31.6)
	Junior NCM	50.3	(47.2, 53.4)	53.4	(50.8, 56.0)	52.2	(50.7, 53.6)
Service element	Army	52.6	(48.8, 56.3)	50.9	(47.5, 54.2)	51.6	(49.1, 54.0)
	Navy	17.3	(14.7, 20.4)	18.3	(16.0, 20.9)	17.9	(16.1, 20.0)
	Air Force	30.1	(26.8, 33.6)	30.8	(27.9, 33.9)	30.5	(28.3, 32.8)
Length of service***	<2 years	F	F	8.4	(6.4, 11.1)	6.3	(4.9, 8.1)
	2–9 years	16.4	(13.4, 19.8)	29.8	(26.6, 33.2)	24.3	(22.1, 26.7)
	10–19 years	20.9	(17.9, 24.2)	11.8	(9.7, 14.1)	15.5	(13.7, 17.4)
	≥20 years	59.5	(55.8, 63.1)	50	(46.9, 53.2)	53.9	(51.6, 56.1)
Health status	Mean SF-12 PCS*	40.4	(39.6, 41.2)	53.8	(53.3, 54.2)	48.3	(47.8, 51.3)
	Mean SF-12 MCS*	47.6	(46.6, 48.6)	52.9	(52.2, 53.5)	50.7	(50.1, 51.3)
	Any physical health condition***	94.3	(92.1, 95.9)	61.3	(57.9, 64.6)	74.9	(72.5, 77.1)
	Mental health problem (moderate/severe)***	63.6	(59.9, 67.0)	31.7	(28.6, 34.9)	44.6	(42.1, 47.1)
	Two or more physical health conditions***	71.5	(67.9, 74.9)	30.4	(27.5, 33.5)	47.3	(44.8, 49.8)
	Comorbid physical health condition and mental health problem***	44.5	(40.8, 48.2)	16.1	(13.6, 18.9)	27.8	(25.5, 30.1)
	Extreme life stress***	6.8	(5.1, 9.2)	2.1	(1.3, 3.3)	4	(3.1, 5.2)
	Suicidal ideation past 12 months***	12.7	(10.5, 15.2)	4.9	(3.6, 6.6)	8.1	(6.8, 9.5)
Adjustment to civilian life***	Easy/very easy	37.3	(33.8, 40.9)	62.8	(59.4, 66.0)	52.4	(49.9. 54.9)
	Neither	16.1	(13.4, 19.1)	14.7	(12.3, 17.4)	15.3	(13.5, 17.2)
	Difficult/very difficult	46.7	(43.0, 50.4)	22.5	(19.8, 25.5)	32.4	(30.1, 34.7)
VAC client in 2016***	Yes	72.6	(68.9, 76.0)	27.2	(24.5, 30.1)	45.7	(43.2, 48.2)
	No	27.4	(24.0, 31.1)	72.8	(69.9, 75.6)	54.3	(51.9, 56.8)

Wt% = weighted percentage of the population in that category; F = estimate too unreliable to be published (Statistics Canada guidelines); Adjustment = ease of adjustment to civilian life; NCM = noncommissioned member. Pearson chi-square test for differences between those with and without pain was significant at *p < .05, **p < .01, or ***p < .001.

### Cluster Characteristics

Table 2 shows the characteristics of the three subgroups of veterans with chronic pain based on the determinant variables used in the cluster analysis. The 47% in cluster I rarely had any of the most severe characteristics in the five determinant variables. The 26% in cluster III commonly had more severe pain, mental health, and activity limitation characteristics. Cluster II (27%) was more like cluster I in most often having high frequencies of low K-10 psychological distress or no PC-PTSD symptom criteria but more like cluster III in having high frequencies of most activities prevented by pain and need for assistance with ADL. The predictor importance analysis indicated that intensity and number of activities limited by pain played lesser roles in the clustering process than the K-10 psychological distress score, number of PTSD symptoms, and degree of activity limitation. The silhouette measure of separation was 0.2, indicating some overlap of the groups owing to heterogeneity in the classification variables.

**Table 2.** Distribution of cluster classification variables among veterans with chronic pain

		Cluster I		Cluster II		Cluster III	
Variable	Category	*n*	%	*n*	%	*n*	%
Total		586	46.8	328	26.9	331	26.2
Pain intensity	Mild	201	34.3	37	11.3	32	9.7
	Moderate	376	64.2	202	61.6	179	54.1
	Severe	9	1.5	89	27.1	120	36.3
Number of activities prevented by pain or discomfort	None	117	20.0	5	1.5	8	2.4
	A few	222	37.9	55	16.8	29	8.8
	Some	179	30.5	116	35.4	88	26.6
	Most	68	11.6	152	46.3	206	62.2
K-10 psychological distress	No/little	480	81.9	223	68.0	0^a^	0.0
	Mild/moderate	99	16.9	87	26.5	125	37.8
	Severe	7	1.2	18	5.5	206	62.2
PTSD symptoms	None	403	68.8	196	59.8	2	0.6
	1–2	144	24.6	77	23.5	39	11.8
	3–4	39	6.7	55	16.8	290	87.6
Activity limitation	None	90	15.4	0^a^	0.0	1	0.3
	HRAL only	496	84.6	29	8.8	92	27.8
	ADL need	0^a^	0.0	299	91.2	238	71.9

Values in the same row not followed by the same are significantly different at *p* < .05 in the test of equality for column proportions within a variable, so two variables in a row that are followed by the same letter are not significantly different.

^a^This category is not used in comparisons because its proportion is equal to zero or one. HRAL only = health-related activity limitations, no ADL assistance needed.

### Socioeconomic and Military Characteristics and Ease of Adjustment to Civilian Life

Table 3 shows that veterans living with chronic pain categorized as cluster I tended to be younger when compared with clusters II and III. Cluster I individuals were less often female compared with cluster II and more often were married and had higher education than cluster III. Cluster I individuals were more often working, had higher military rank, and less likely to report difficult adjustment to civilian life. Cluster II individuals were more often women than the other two clusters. Cluster III individuals were more often middle-aged, were less often married, more often had lower education, were more often not working, were much more often on disability, had junior noncommissioned rank, had middle years of service, and reported a difficult adjustment to civilian life. Cluster III individuals were more often middle-aged, single, or never married when compared with the other two clusters; more often had lower education; were not working; were much more often on disability; had junior noncommissioned rank; had middle years of service; and had a difficult adjustment to civilian life.

**Table 3.** Distribution of socioeconomic and military characteristics among veterans with chronic pain, by cluster (unweighted data)

		Cluster I		Cluster II		Cluster III	
Variable	Category	*n*	%	*n*	%	*n*	%
Age	<30	29	4.9	5	1.5	5	1.5
	30–39	54	9.2	25	7.6	56	16.9
	40–49	104	17.7	68	20.7	95	28.7
	50–59	276	47.1	161	49.1	137	41.4
	60+	123	21.0	69	21.0	38	11.5
Sex	Male	523	89.2	254	77.4	285	86.1
	Female	63	10.8	74	22.6	46	13.9
Marital status	Married/common law	488	83.3	264	80.5	241	73.0
	Separated/widowed/divorced	49	8.4	40	12.2	44	13.3
	Single/never married	49	8.4	24	7.3	45	13.6
Education	High school or less	233	39.8	142	43.8	159	48.0
	Postsecondary < bachelor	210	35.8	119	36.7	122	36.9
	Postsecondary ≥ bachelor	143	24.4	63	19.4	50	15.1
Household income	$0 to <$50,000	59	10.5	37	11.9	62	20.3
	$50,000 to <$100,000	222	39.5	142	45.7	140	45.9
	$100,000 to <$150,000	145	25.8	84	27.0	67	22.0
	$150,000+	136	24.2	48	15.4	36	11.8
Satisfaction with finances	Satisfied	427	73.1	214	65.4	143	43.2
	Neither	69	11.8	50	15.3	70	21.1
	Dissatisfied	88	15.1	63	19.3	118	35.6
Main activity prior year	Worked in job or reserves	348	59.4	128	39.0	92	28.0
	Retired, not looking for work	140	23.9	84	25.6	51	15.5
	Attended school or training	35	6.0	23	7.0	31	9.5
	Looked for work	27	4.6	11	3.4	12	3.7
	Cared for family member	14	2.4	10	3.0	5	1.5
	Disabled or on disability	22	3.8	72	22.0	137	41.8
Rank	Officer	182	31.1	90	27.4	64	19.3
	Senior NCM	237	40.4	141	43.0	131	39.6
	Junior NCM	167	28.5	97	29.6	136	41.1
Element	Navy	110	18.8	61	18.6	42	12.7
	Army	285	48.6	151	46.0	205	61.9
	Air Force	191	32.6	116	35.4	84	25.4
Length of service	<2 years	13	2.2	1	0.3	5	1.5
	2–9 years	80	13.7	30	9.2	48	14.5
	10–19 years	79	13.5	70	21.4	94	28.4
	≥20 years	414	70.6	226	69.1	184	55.6
Adjustment to civilian life	Easy	318	54.4	127	38.7	36	10.9
	Neither	97	16.6	62	18.9	22	6.6
	Difficult	170	29.1	139	42.4	273	82.5

Values in the same row not followed by the same are significantly different at *p* < .05 in the test of equality for column proportions within a variable, so two variables in a row that are followed by the same letter are not significantly different. NCM = noncommissioned member; Adjustment = ease of adjustment to civilian life.

### Health Indicators

Table 4 shows differences between the clusters in comorbidity combinations, mental health problems, life stress, suicidal ideation, and SF-12 PCS and MCS scores. Very few in all clusters had none of the chronic physical health conditions asked about in the survey. Comorbidity of physical conditions and mental health problems increased across the clusters. Almost all participants in cluster III had severe mental health problems, to a much greater degree than those in clusters I or II. Quite a bit or extreme life stress and past-year suicidal ideation were also much more common in cluster III. Both SF-12 MCS and PCS score differences between clusters were significant. MCS and PCS median scores were below the population means for all clusters, but mental health-related quality of life was markedly diminished in cluster III, and physical health-related quality of life was markedly diminished in both clusters II and III.

**Table 4.** Distribution of health characteristics among veterans with chronic pain, by cluster (unweighted data)

		Cluster I		Cluster II		Cluster III	
Variable	Category	*n*	%	*n*	%	*n*	%
Number of physical health conditions	0	46	7.9	11	3.4	7	2.1
	1	162	27.7	58	17.7	40	12.1
	2	170	29.1	98	30.0	84	25.5
	≥3	206	35.3	160	48.9	199	60.3
Mental health problems	No/little	333	56.8	133	40.5	01	0.0
	Moderate	207	35.3	122	37.2	15	4.5
	Severe	46	7.8	73	22.3	315	95.5
Comorbidity of physical health conditions and mental health problems	No PHC, no MHP	32	5.5	3	0.9	0^a^	0.0
	PHC, no MHP	300	51.4	130	39.8	0^a^	0.0
	MHP, no PHC	14	2.4	8	2.4	7	2.1
	PHC and MHP	238	40.8	186	56.9	322	97.9
Life stress	Not at all/not very	215	36.7	93	28.5	18	5.4
	A bit	263	44.9	161	49.4	92	27.8
	Quite a bit/extremely	108	18.4	72	22.1	221	66.8
Suicidal ideation prior year	Yes	23	3.9	20	6.1	134	41.1
	No	561	96.1	307	93.9	192	58.9

Values in the same row not followed by the same are significantly different at *p* < .05 in the test of equality for column proportions within a variable, so two variables in a row that are followed by the same letter are not significantly different. ^a^This category is not used in comparisons because its proportion is equal to zero or one.

PHC = physical health condition; MHP, mental health problem.

**Figure 2.**
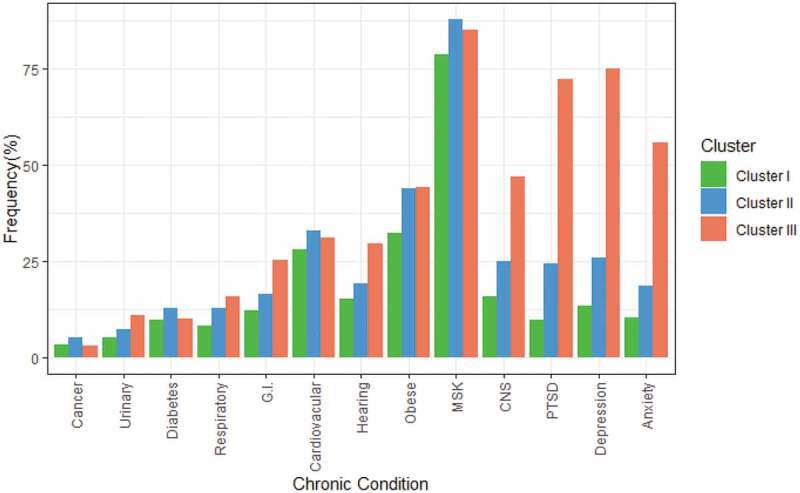
Frequencies of chronic health conditions among veterans with chronic pain, by cluster (unweighted data). Anxiety = anxiety disorder such as phobia, obsessive–compulsive disorder, or panic disorder; Depression = mood disorder such as depression, mania, dysthymia, or bipolar disorder; CNS = central nervous system (migraine, Alzheimer’s or other dementia, or effects of a traumatic brain injury or concussion); MSK = muskuloskeletal (arthritis or back problems, excluding fibromyalgia); Hearing = hearing impairment; Cardiovascular = heart disease, effects of a stroke or high blood pressure; G.I. = gastrointestinal (bowel disorder such as Crohn’s, ulcerative colitis, irritable bowel, or bowel incontinence or intestinal or stomach ulcers); Urinary = urinary incontinence; Respiratory = asthma, chronic bronchitis, emphysema or chronic obstructive pulmonary disease

Figure 2 compares clusters by chronic health conditions. A considerable majority in all three clusters (more than three-quarters) reported musculoskeletal conditions. Veterans in cluster I least often had the chronic physical or mental health conditions asked about in the survey. Veterans in cluster III much more often had chronic mental health conditions and more often had gastrointestinal, central nervous system, or hearing problems.

### Social Support and Service Utilization Indicators

Table 5 demonstrates that veterans in cluster III more often reported low perceived social support. All veterans with chronic pain had seen a family doctor in the prior year, and about a fifth of those in clusters I and II had ten or more family doctor visits. Veterans in cluster III more often had high family doctor utilization (ten or more visits), had spoken to a health professional about mental health problems, or had an overnight hospital stay. A majority in all three clusters were VAC clients, but nearly all in cluster III (95%) were receiving benefits and services from VAC.

**Table 5.** Distribution of social support and service utilization characteristics among veterans with chronic pain, by cluster (unweighted data)

		Cluster I		Cluster II		Cluster III	
Variable	Category	*n*	%	*n*	%	*n*	%
Perceived social support	Low (≤29)	65	11.3	51	16.0	163	52.8
	High (>29)	508	88.7	267	84.0	146	47.2
Number of family doctor visits in past 12 months	0	0^a^	0.0	0^a^	0.0	0^a^	0.0
	1–3	325	55.5	134	40.9	97	29.3
	4–9	124	21.2	129	39.3	122	36.9
	10+	137	23.4	65	19.8	112	33.8
Saw or talked to health professional about emotional/mental health in past 12 months	Yes	99	16.9	115	35.1	254	76.7
	No	487	83.1	213	64.9	77	23.3
Patient in hospital overnight in past 12 months	Yes	32	5.5	43	13.1	66	19.9
	No	554	94.5	285	86.9	265	80.1
VAC client in 2016	Yes	363	61.9	291	88.7	315	95.2
	No	223	38.1	37	11.3	16	4.8

Values in the same row not followed by the same are significantly different at *p* < .05 in the test of equality for column proportions within a variable, so two variables in a row that are followed by the same letter are not significantly different. ^a^This category is not used in comparisons because its proportion is equal to zero or one.

## Discussion

**Figure 3.**
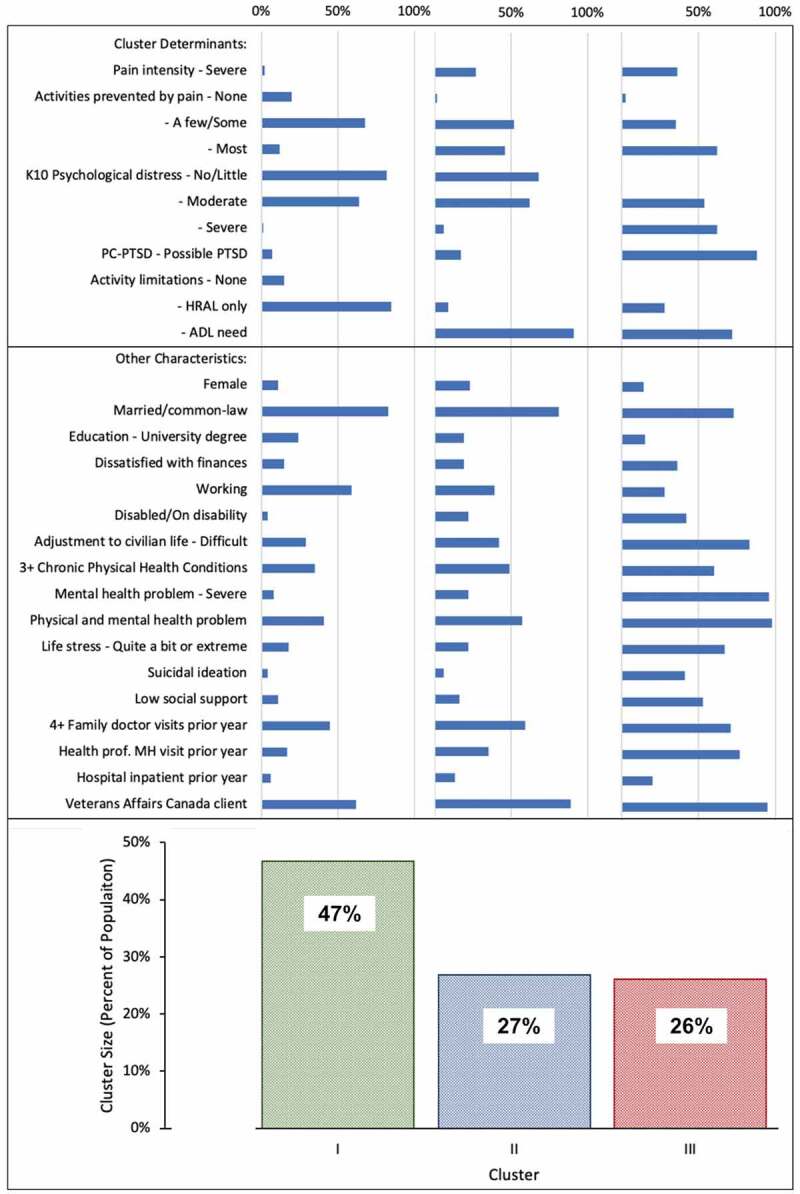
Summary of cluster size (percentage of those with pain) and characteristics (percentage of those in cluster). HRAL only = health-related activity limitations, no ADL assistance needed; MH = mental health

CAF Regular Force veterans with chronic pain (41% of those released between 1998 and 2015 and surveyed in 2016) showed high variability in terms of socioeconomic, military, health, social integration, and service utilization characteristics. Using pain characteristics, mental health, and activity limitation indicators, this exploratory cluster analysis combined with assessment of cluster characteristics did not identify unique subgroups of veterans with chronic pain owing to heterogeneity among individuals. However, the analysis did produce an overview of the pain population (Figure 3). A majority of the largest cluster (cluster I, 47%) generally were doing well as indicated by low frequencies of severe pain, most activities prevented by pain, severe psychological distress or PTSD symptomatology, and need for assistance with activities of daily living. The remaining 53% were in two clusters characterized by higher frequencies in more adverse categories. Cluster III (26% of the pain population) generally had the highest frequencies of adverse indicators and service utilization. There were individuals in all variable categories in all three clusters, reflecting the heterogeneity among Canadian veterans living with chronic pain.

The finding of high heterogeneity in veterans with chronic pain is consistent with findings from cluster analysis attempts in general populations. We found no other cluster analysis reports in military veterans, but there were similar studies using varying combinations of pain characteristics, mental health status, functioning, or quality of life measures in others. The studies identified varying numbers of nonunique clusters. A Spanish adult general population study identified two nonunique clusters based on pain characteristics.^40^ One cluster more often had shorter duration pain in one site and the other tended to have longer duration pain in more than one site. Anxiety and concerns about impacts on family were more common in the latter cluster. A Swedish study of older adults identified four nonunique clusters characterized by pain intensity, number of pain sites, and degree of psychological symptoms.^41^ One of these clusters (33%) had indicators of generally doing better than the others. Poor mental health characterized the other three clusters irrespective of pain intensity. A Swedish study of patients with chronic pain visiting multidisciplinary pain center identified four nonunique clusters based on pain characteristics, mental health, and quality of life measures in an attempt to identify subgroups for tailoring care to match needs.^42^ The clusters were characterized in general by differences in mental health, pain characteristics, and social factors. One cluster (28%) had the lowest levels of mental health problems and severe pain characteristics. A U.S. study of patients with chronic pain in treatment at a pain center distinguished three nonunique clusters based on psychological distress, pain interference with activities, and pain behaviors.^44^ Their clusters were similar in demographic characteristics, compensation status, pain duration, and pain intensity. As in other studies, the clusters differed in severity of psychological distress. A cluster analysis of members of a U.S. health maintenance organization with two or more chronic conditions identified ten clusters dominated by different conditions. One cluster was dominated by patients with chronic pain, a majority of whom had mental health conditions, and about half had obesity.^45^ A German study of patients with low back pain in primary care identified four clusters based on employment, age, pain characteristics, mental health, and quality of life.^46^ Patients in the two clusters with the highest costs per patient more often had mental health problems. These studies demonstrate that cluster analysis does not identify unique subgroups of people with chronic pain but does help to paint a picture of chronic pain populations in ways that can inform service planning.

The heterogeneity findings support calls for person-centered care that can match individuals’ needs for specific types and intensity of services.^2,18,24^ Although we cannot draw conclusions about causality from cross-sectional data, it is well established that the experience and impact of chronic pain in individuals’ lives is attributed to a variety of biological, developmental, and environmental factors across the life course, including adverse childhood experiences.^2,6,18,42,47,48^ Stepped, stratified, and matched chronic pain care models have been proposed to address individual needs for health care and supports in any of the well-being domains in ways that efficiently utilize effective support services.^2,18,22^ The finding that veterans in cluster I (nearly half of the veterans with chronic pain) much less often had high degrees of physical health multimorbidity, mental health problems, extreme life stress, and suicidal ideation suggests that most are unlikely to need the highly specialized services of chronic pain centers. However, that finding does not suggest that they would not benefit from interdisciplinary care, and some likely would benefit from specialized care early on to prevent progression into more complex states.^22,24^ The finding that half of those with chronic pain were in clusters II and III suggests the extent of the need for specialized interdisciplinary services for these veterans.

The findings that all veterans with chronic pain had visited family physicians in the year prior to the survey and that the proportion with high use of family physicians was higher in clusters with more complex features demonstrate the importance of accounting for primary care in designing chronic pain care systems along with other types of community services.^2,18,21,49^ Chronic pain is common in primary care and is challenging to manage in that setting.^11,21,49^ The VHA established a person-centered stepped care model with integrated primary care that has evidence of effectiveness in that environment, where health care is delivered in VHA clinical facilities.^21,23,49^ Chronic pain is common among veterans participating in VAC programs, and in this study a majority in all three clusters were reached by VAC programs. Though VAC programs provide support for well-being in multiple domains, veterans in Canada obtain health care services from providers in public and private health care systems.

Our findings point to the value of addressing multiple well-being domains, including mental health, when supporting veterans with chronic pain. Two of the five cluster determinant variables were mental health indicators, so it is not surprising that the clusters were strongly distinguished by mental health status. However, the cluster analysis demonstrated both the degree to which mental health problems were present in these veterans and that indicators of poor well-being in other domains were more common in the one-quarter (cluster III) with high frequencies of mental health problems (Figure 3). There is substantial evidence of the importance of addressing mental health and well-being in other domains in veterans with chronic pain.^2,5,6,11,18,23,24,27,47,50^ Determinants of mental health arise from multiple well-being domains and, conversely, adverse mental health can influence pain experiences and well-being in multiple aspects of life.^6,11,19,47^

The finding that most veterans in cluster II needed assistance with ADL and few had severe psychological distress is of interest. Similarly, though very few in cluster I had severe mental health problems and none had ADL need, most had health-related activity limitations. In LASS surveys, a majority (95%) of those with chronic mental health conditions had physical health conditions, whereas only 28% with physical conditions had mental conditions.^20^ These findings could be explained by several hypotheses. Many with chronic pain-related activity limitations could be doing well psychologically because their well-being needs in other domains are being met, such as having a vocation, sufficient finances, good social integration and support, good housing, and adequate health care treatment.^47^ Some might have been more resilient to developing mental health problems and had adapted well to living with chronic pain.

### Strengths and Limitations

Strengths of this study include confirming veteran status and military characteristics through data linkage rather than relying on self-report of veteran status. The data linkage enabled the development of a total population sample frame from which to draw a representative sample. The sample size is statistically representative of CAF Regular Force veterans released between 1998 and 2015. The weighting procedure used by Statistics Canada adjusts for nonresponse. Finally, the survey was conducted by Statistics Canada independent of VAC and CAF to ensure veteran privacy and encourage participation.

Limitations include an inability to establish the direction of associations due the cross-sectional design of our study; specifically, we cannot be certain whether chronic pain is a cause or effect of associated factors (e.g., financial stress, greater physical and mental health problems, extreme life stress, suicidal ideation, and difficult adjustment to civilian life). It is not known whether the unspecified duration of the HUI3 chronic pain survey question could overestimate chronic pain prevalence compared to studies that use a 3+-month minimum. Though LASS survey findings suggest that the HUI3 module seems to underestimate the prevalence of chronic pain based on asking directly whether the respondent has chronic pain or discomfort,^26^ a systematic review suggested that survey methods could overestimate chronic pain prevalence compared to interview assessment.^1^ However, the same HUI3 module is used in general population studies, allowing overall prevalence comparison. The cluster analysis methodology used in this study used unweighted data. However, this would not affect the relevance of the characteristics identified. The LASS surveys did not include addiction and substance use indicators owing to survey length limitations. The findings are male-centric because the data set contained relatively few women owing to the lower proportion of women in military service. Regular Force veterans released since 1998 may differ from veterans released in prior eras. Although Reserve Force veterans were not included in the LASS 2016 survey, prior LASS surveys found that reservists who deployed in support of operations had characteristics similar to those of Regular Force members.^26,51^

### Implications

The finding that chronic pain is common among CAF veterans with a high degree of heterogeneity has implications for policy and services. Chronic pain prevention has long been recognized by the military as an important challenge.^9^ Though our study was not able to map the types of chronic pain care that veterans receive, the findings support calls for person-centered care capable of addressing physical and mental health as well as well-being in other areas of life.^2^ The findings support the VAC’s well-being policy approach of addressing both access to mental health services and support for determinants of mental health across multiple well-being domains.^17,26^ Though chronic pain system development initiatives are under way across Canadian communities, systematic barriers need to be overcome to support the development of widely accessible person-centered, multidisciplinary and collaborative care.^2,18,21–24^

There is need for research that maps and evaluates the effectiveness of individual pathways to good well-being for CAF veterans with chronic pain.^2,18,24,49^ Though there are some observational data about outcomes for CAF veterans in least one specialized pain center,^50^ little is known about the nature and effectiveness of health care and supports received by most CAF veterans with chronic pain. As in the United States,^23^ further research is needed to identify existing pathways for CAF veterans with chronic pain, explore triage strategies based on veterans’ individual needs, and conduct clinical trials to assess the effectiveness of integrated person-centered, multidisciplinary care models. Future research is also required to establish factors associated with the development of chronic pain, to inform the direction of the association. Study of those with pain-related activity limitations but good mental health could yield useful information for treatment. Finally, little is known about chronic pain experiences in certain subgroups, such as women and aboriginal veterans.
